# Physical Activity and Sedentary Behavior in Metabolically Healthy versus Unhealthy Obese and Non-Obese Individuals – The Maastricht Study

**DOI:** 10.1371/journal.pone.0154358

**Published:** 2016-05-03

**Authors:** Belle H. de Rooij, Julianne D. van der Berg, Carla J. H. van der Kallen, Miranda T. Schram, Hans H. C. M. Savelberg, Nicolaas C. Schaper, Pieter C. Dagnelie, Ronald M. A. Henry, Abraham A. Kroon, Coen D. A. Stehouwer, Annemarie Koster

**Affiliations:** 1 Department of Social medicine, Maastricht University, Maastricht, the Netherlands; 2 CAPHRI School for Public Health and Primary Care, Maastricht University, Maastricht, the Netherlands; 3 Department of Internal Medicine, Maastricht University Medical Centre (MUMC+), Maastricht, the Netherlands; 4 Cardiovascular Research Institute Maastricht (CARIM), Maastricht University Medical Centre, Maastricht, the Netherlands; 5 Department of Human Movement Sciences, Maastricht University, Maastricht, the Netherlands; 6 School for Nutrition and Translational Research in Metabolism (NUTRIM), Maastricht University, Maastricht, the Netherlands; 7 Department of Epidemiology, Maastricht University, Maastricht, the Netherlands; GDC, GERMANY

## Abstract

**Background:**

Both obesity and the metabolic syndrome are associated with increased risk of cardiovascular diseases and type 2 diabetes. Although both frequently occur together in the same individual, obesity and the metabolic syndrome can also develop independently from each other. The (patho)physiology of “metabolically healthy obese” (i.e. obese without metabolic syndrome) and “metabolically unhealthy non-obese” phenotypes (i.e. non-obese with metabolic syndrome) is not fully understood, but physical activity and sedentary behavior may play a role.

**Objective:**

To examine objectively measured physical activity and sedentary behavior across four groups: I) “metabolically healthy obese” (MHO); II) “metabolically unhealthy obese” (MUO); III)”metabolically healthy non-obese” (MHNO); and IV) “metabolically unhealthy non-obese” (MUNO).

**Methods:**

Data were available from 2,449 men and women aged 40–75 years who participated in The Maastricht Study from 2010 to 2013. Participants were classified into the four groups according to obesity (BMI≥30kg/m^2^) and metabolic syndrome (ATPIII definition). Daily activity was measured for 7 days with the activPAL physical activity monitor and classified as time spent sitting, standing, and stepping.

**Results:**

In our study population, 562 individuals were obese. 19.4% of the obese individuals and 72.7% of the non-obese individuals was metabolically healthy. After adjustments for age, sex, educational level, smoking, alcohol use, waking time, T2DM, history of CVD and mobility limitation, MHO (n = 107) spent, per day, more time stepping (118.2 versus 105.2 min; p<0.01) and less time sedentary (563.5 versus 593.0 min., p = 0.02) than MUO (n = 440). In parallel, MHNO (n = 1384) spent more time stepping (125.0 versus 115.4 min; p<0.01) and less time sedentary (553.3 versus 576.6 min., p<0.01) than MUNO (n = 518).

**Conclusion:**

Overall, the metabolically healthy groups were less sedentary and more physically active than the metabolically unhealthy groups. Therefore, physical activity and sedentary time may partly explain the presence of the metabolic syndrome in obese as well as non-obese individuals.

## Introduction

Obesity is a worldwide growing health concern that has been associated with numerous poor health outcomes, such as type 2 diabetes (T2DM), cardiovascular disease (CVD), arthritis, certain types of cancer and mortality [1]. Obesity often clusters with risk factors of CVD and T2DM that comprise the metabolic syndrome (MetS), including insulin resistance, hypertension, dyslipidemia and low levels of HDL-cholesterol [2]. However, an obese group has been identified that does not present MetS, the so-called “metabolically healthy obese” [3]. A recent review shows that this group, about one third of all obese individuals, has a CVD risk that is lower compared to their metabolically unhealthy counterparts and only slightly increased compared to that of non-obese individuals [4]. Conversely, a group of non-obese individuals presents MetS and is at increased cardio-metabolic risk, the “metabolically unhealthy non-obese” [5]. Based on the assumption that obesity and poor metabolic health do not always occur together, we can distinguish four phenotypes: I) metabolically healthy obese (MHO), II) metabolically unhealthy obese (MUO), III) metabolically healthy non-obese (MHNO) and IV) metabolically unhealthy non-obese (MUNO). Factors that explain differences between MHO, MUO, MHNO and MUNO are not fully understood. In particular, little is known about the role of lifestyle factors such as physical activity and sedentary behavior on the presence of MetS in obese and non-obese individuals.

Previous research has identified low levels of moderate to vigorous physical activity (MVPA) as an important predictor of MetS [6–8]. In addition to MVPA, sedentary behavior has been identified as a distinct health behavior that differs from a lack of MVPA and this behavior has recently been associated with adverse health effects including MetS [9–12]. Sedentary behavior is defined as waking activities performed with low energy expenditure and involves sitting or lying down, computer use, TV watching or sitting in a car [13]. Additionally, the pattern in which physical activity and sedentary behavior are accumulated seems to be important. Previous studies have for example shown that interruptions in sedentary time are associated with a more favorable metabolic risk profile [9,14–17]. To date, the differences in physical activity and sedentary behavior have not been investigated extensively in MHO, MUO, MHNO and MUNO. Physical activity and sedentary behavior could potentially explain the presence of MetS in obese and non-obese individuals, but may as well explain the differences between obese and non-obese individuals within metabolically healthy and unhealthy populations.

Existing evidence for the role of physical activity and sedentary behavior mostly comprises self-reported physical activity and sedentary behavior data, and findings are conflicting. As expected, some studies found higher levels of self-reported physical activity in MHO compared to MUO [3,13,18–21] and higher in MHNO compared to MUNO [3,19,22]. Another study reported lower self-reported sedentary time in MHNO compared to MUNO [23]. To date, only few studies have objectively examined physical activity across metabolically healthy and unhealthy groups. Poelkens and colleagues [4] found a higher total step count in MHO compared to MUO, but others did not find any significant differences in step count or total energy expenditure between these groups [24,25]. Recently, Bell and colleagues [26] objectively examined physical activity patterns by wrist-worn accelerometry in healthy and unhealthy obese, overweight and normal weight individuals. They found that MHO were overall more physically active compared to MUO and comparable physically active to the unhealthy non-obese groups. However, MHO and MUO did not significantly differ in time spent in MVPA. Our study aimed to examine how MHO, MUO, MHNO and MUNO differ with respect to objectively measured physical activity and sedentary behavior measured on seven days in The Maastricht Study.

## Materials and Methods

### Study design and population

We used data from The Maastricht Study, an observational prospective population-based cohort study. The rationale and methodology have been described previously [27]. In brief, the study focuses on the etiology, pathophysiology, classic complications, such as cardiovascular disease, retinopathy, neuropathy and nephropathy and of emerging comorbidities, such as cognitive decline, depression, and gastrointestinal, musculoskeletal and respiratory diseases of T2DM and is characterized by an extensive phenotyping approach. Eligible for participation were all individuals aged between 40 and 75 years and living in the southern part of the Netherlands. Participants were recruited through mass media campaigns and from the municipal registries and the regional Diabetes Patient Registry via mailings. Recruitment was stratified according to known T2DM status, with an oversampling of individuals with T2DM, for reasons of efficiency. The present report included cross-sectional data from a selection of 3,451 participants, who completed the baseline survey between September 2010 and October 2013. 67 Participants with type 1 diabetes or a BMI lower than 18.5 kg/m^2^ were excluded from analyses. activPAL^tm^ data were not available from 796 participants, partly because the activPAL was later added to the study protocol and because of logistic reasons. Covariates were missing from 139 participants. A total number of 2,449 participants were included in our analysis. The examinations of each participant were performed within a time window of three months. The study has been approved by the institutional medical ethical committee (NL31329.068.10) and the Minister of Health, Welfare and Sports of the Netherlands (Permit 131088-105234-PG). All participants gave written informed consent.

### Measures

#### Obesity

Weight and height were measured during the first visit at the research center in the morning, without shoes and wearing light clothing using a scale and stadiometer to the nearest 0.5 kg or 0.1 cm (Seca, Hamburg, Germany). Body Mass Index (BMI) was calculated as weight in kg divided by height in meters squared. Obesity was defined as a BMI greater than or equal to 30 kg/m^2^ and the obese and non-obese groups were classified accordingly.

#### Metabolic syndrome

Measures that were used to define MetS included waist circumference, triglycerides, high-density lipoprotein (HDL) cholesterol, diastolic and systolic blood pressure, fasting plasma glucose and medication use. Waist circumference was measured with a flexible plastic tape measure (Seca, Hamburg, Germany), in duplicate midway between the lower rib margin and the iliac crest at the end of expiration, to the nearest 0.5 cm. Fasting blood samples were collected and laboratory assessments were conducted of fasting levels of glucose, HDL-cholesterol and serum triglyceride level. Plasma glucose was measured with a standard enzymatic hexokinase reference method, and HDL cholesterol and triglycerides levels were measured with standard (enzymatic and/or colorimetric) methods by an automatic analyzer (Beckman Synchron LX20, Beckman Coulter Inc., Brea, USA; or Roche Cobas 6000, Roche diagnostics, Mannheim, Germany). Office blood pressure was measured three times on the right arm after a 10-minute rest period, using a non-invasive blood pressure monitor (Omron 705IT, Japan). The blood pressure cuff was secured approximately 2 cm above the elbow joint. When the difference between measurement two and three was more than 10 mmHg, a fourth measurement was performed. All available measurements were used to calculate the average blood pressure. Ambulatory 24-h blood pressure (WatchBP O3, Microlife, Switzerland) was measured at the non-dominant arm using an ambulatory device that is programmed to take blood pressure readings every 15 minutes from 8.00–23.00 and every 30 minutes from 23.00–8.00. During a medication interview generic name, dose and frequency are registered by trained staff. Additionally, participants were requested to bring all medication they use or a list from their pharmacists to the research center.

MetS was defined according to the Adult Treatment Panel III definition [2], meeting at least three out of the following criteria: (I) waist circumference ≥102 cm in men and ≥88 cm in women; (II) serum triglyceride level ≥150 mg/dl (≥1.7 mmol/L) or on current drug treatment for high triglycerides; (III)HDL-Cholesterol level <40 mg/dl (<1.0 mmol/L) in men and <50 mg/dl (<1.3 mmol/L) in women; (IV) diastolic blood pressure ≥85 mm Hg and/or systolic blood pressure ≥130 mm Hg or using antihypertensive medications; and (V) fasting glucose level ≥100 mg/dl (≥5.6 mmol/L) and/or using anti-diabetic medication[2]. Office blood pressure was used to define MetS in our main analysis.

#### Physical activity and sedentary behavior

The activPAL physical activity monitor (PAL Technologies Limited, Glasgow, Scotland, UK) was used to objectively measure daily activity patterns on multiple days. The activPAL is a lightweight (15 g), small (53x35x7 mm) triaxial accelerometer that records movement in three planes (vertical, anterior-posterior and mediolateral), and also determines posture (sitting or lying, standing and stepping) based on acceleration information. The device was attached directly to the skin on the front of the right thigh, with transparent 3M Tegaderm tape, after the device had been waterproofed using a nitrile sleeve. Participants were asked to wear the accelerometer for eight consecutive days, without removing the device at any time. To avoid inaccurately identifying non-wear time, participants were asked not to replace the device once removed. Data were uploaded using the activPAL software and processed using customized software written in MATLAB® R2013b (MathWorks. Natick, MA, USA) in which wake and bed times were automatically detected to estimate total waking time [12,28]. Data of the first day were excluded from analysis because participants performed physical function tests at the research center after the device was attached. In addition, data from the final wear day providing ≤ 14 waking hours of data were excluded from the analysis. Participants were included if they provided at least 1 valid day (≥10 h of waking data).

Based on acceleration information, posture was determined and the total amount of time (min/day) spent sedentary (sitting/lying), standing and stepping were calculated during waking time. Stepping intensity was determined as the number of steps per minute. Stepping time was divided into low intensity stepping time (minutes with a step frequency ≤110 steps/min) and high intensity stepping time (minutes with a step frequency >110 steps/min) [29]. In this study we used high intensity stepping time as a measure for MVPA, which has earlier shown to be comparable to MVPA [29]. Further, proportional time spent sedentary, standing, and stepping (low and high intensity) were calculated as a percentage of total waking time on valid days. Sedentary breaks were defined as any interruption of a sedentary bout by standing or stepping. The mean daily number of sedentary breaks was calculated.

#### Covariates

Covariates were age, sex, educational level, BMI, smoking, alcohol use, T2DM and CVD. Educational level was measured on an eight-point scale ranging from 1 (no education) to 8 (university education) and was classified as low (no education, primary education not completed, primary education, lower vocational education), medium (intermediate vocational education, higher secondary education), or high (higher professional education, university education). Smoking status was classified into ‘never smoker’, ‘former smoker’ or ‘current smoker’. Alcohol use was classified into ‘no alcohol use’, ‘low alcohol use’ (women ≤7 glasses per week, men ≤14 glasses per week) or ‘high alcohol use’ (women >7 glasses per week, men >14 glasses per week). T2DM was assessed by fasting glucose levels (cut-off values of ≥126 mg/dl (7.0 mmol/L)) and 2-hours oral glucose tolerance test (cut-off values of≥200 mg/dl (11.1mmol/L) and diabetic medication use (i.e. oral glucose lowering medication and insulin) CVD status was a dichotomous measure of no history of CVD or a history of CVD defined according to the Rose questionnaire [19]. Mobility limitation was obtained from the EuroQol-5D questionnaire and was defined as having any difficulties with walking in the previous week.

### Statistical analysis

Statistical analysis was conducted with IBM SPSS Statistics for Windows, version 22.0 (IBM Corp, Armonk, NY, USA). To examine the baseline characteristics between obese with and without MetS and non-obese with and without MetS (MHO versus MUO, and MHNO versus MUNO), Chi^-^square tests were used for categorical variables, t-tests for normally distributed continuous variables and the Mann-Whitney U Test for not normally distributed continuous variables. We visually inspected the distribution of each parameter using normal probability plots. General linear models were conducted to compare sedentary behavior (sedentary time, sedentary break count) and physical activity (standing time, high intensity and low intensity stepping time) across the four groups. High intensity stepping time was not normally distributed and was therefore logarithmically transformed. The first model was adjusted for age, sex, educational level, smoking, alcohol use and waking time. The second model was additionally adjusted for T2DM status, history of CVD and mobility limitation. Analysis of mean number of sedentary breaks per day was additionally adjusted for sedentary time. To examine the association with sedentary behavior independent from level of physical activity, the third model was additionally adjusted for high intensity stepping time (step frequency >110 steps/min). Bonferroni-corrected pairwise comparisons were made between the four groups. All statistical contrasts were made at the .05 level of significance.

## Results

In our study population, 547 individuals were obese and 1,902 individuals were non-obese, of whom 19.6% (n = 107) and 72.8% (n = 1,384) were metabolically healthy in the obese and non-obese individuals respectively. The obese individuals had a lower educational level and a lower alcohol use compared to the non-obese. On average, the metabolically healthy obese (MHO) and the metabolically healthy non-obese (MHNO) were younger, had a higher BMI and were more likely to be female and non-smoker compared their metabolically unhealthy counterparts (Table 1).

**Table 1:** Baseline characteristics of the four groups with and without obesity and the metabolic Syndrome.

		Obese			Non-obese		
	Total	MHO	MUO	P-value	MHNO	MUNO	P-value
**N**	2449	107	440		1384	518	
**Age (years), mean±SD**	60.0±8.1	56.5±8.7	61.5±7.9	<0.01	59.1±8.1	62.0±7.7	<0.01
**Sex, %**							
men	52.2	31.8	65.0	<0.01	45.7	63.1	<0.01
women	47.8	68.2	35.0		54.3	36.9	
**Education, %**							
low	33.8	42.1	48.9	0.43	27.0	37.3	<0.01
medium	28.1	29.9	27.5		28.1	28.0	
high	38.2	28.0	23.6		44.9	34.7	
**Smoker, %**							
never	35.1	37.4	27.0	0.03	39.7	28.9	<0.01
former	52.0	55.1	58.0		49.4	53.2	
current	12.9	7.5	15.0		11.0	18.1	
**Alcohol use, %**							
no	18.1	26.2	27.3	0.47	14.2	18.9	<0.03
low	56.6	58.9	53.2		57.6	56.6	
high	25.3	15.0	19.5		28.2	24.5	
**Type 2 diabetes, %**	28.7	17.8	64.8	<0.01	10.3	49.6	<0.01
**History of CVD, %**	16.6	13.1	25.0	<0.01	13.3	18.9	<0.01
**Mobility limitation, %**							
No limitations	83.4	80.4	66.1	<0.01	90.4	80.1	<0.01
Problems walking	16.5	19.6	33.9		9.5	19.7	
**BMI (kg/m**^**2**^**), mean ±SD**	27.1±4.5	32.6±2.8	33.8±3.6	<0.01	24.5±2.4	27.1±2.0	<0.01
**SBP (mmHG), mean±SD**	135.3±18.3	131.5±18.3	142.3±16.4	<0.01	130.6±17.8	142.6±16.9	<0.01
**DBP (mmHG), mean±SD**	76.4±9.8	77.7±8.9	79.2±9.8	0.14	74.5±9.4	78.9±10.0	<0.01
**Triglycerides (mmol/L), median(25**^**th**^**-75**^**th**^**)**	1.2 (0.9–1.7)	1.2 (1.0–1.4)	1.8 (1.3–2.2)	<0.01	1.0 (0.8–1.3)	1.8 (1.3–2.4)	<0.01
**HDL-C (mmol/L), mean±SD**							
Men	1.4±0.4	1.4±0.3	1.2±0.3	<0.01	1.5±0.4	1.2±0.3	<0.01
Women	1.8±0.5	1.7±0.4	1.4±0.4		1.9±0.5	1.5±0.4	
**Glucose (mmol/L), median(25**^**th**^**-75**^**th**^**)**	5.5 (5.1–6.6)	5.3 (5.0–5.5)	6.9 (6.0–8.2)	<0.01	5.2 (4.9–5.6)	6.2 (5.7–7.6)	<0.01
**Waist circumference (cm), mean ±SD**							
Men	101.5±12.4	110.6±8.6	117.4±9.8	<0.01	93.3±6.6	102.5±7.1	<0.01
Women	89.8±12.4	103.5±9.5	109.2±10.8	<0.01	83.5±7.6	94.0±6.6	<0.01
**Total wear time (days), mean±SD**	6.3±1.0	6.3±1.1	6.1±1.2	0.27	6.4±1.0	6.3±1.0	0.05
**Mean daily waking time (minutes), mean±SD**	942.7±54.5	932.6±60.3	938.2±63.5	0.41	945.7±51.1	941.2 ±53.4	0.09

**Fig 1:**
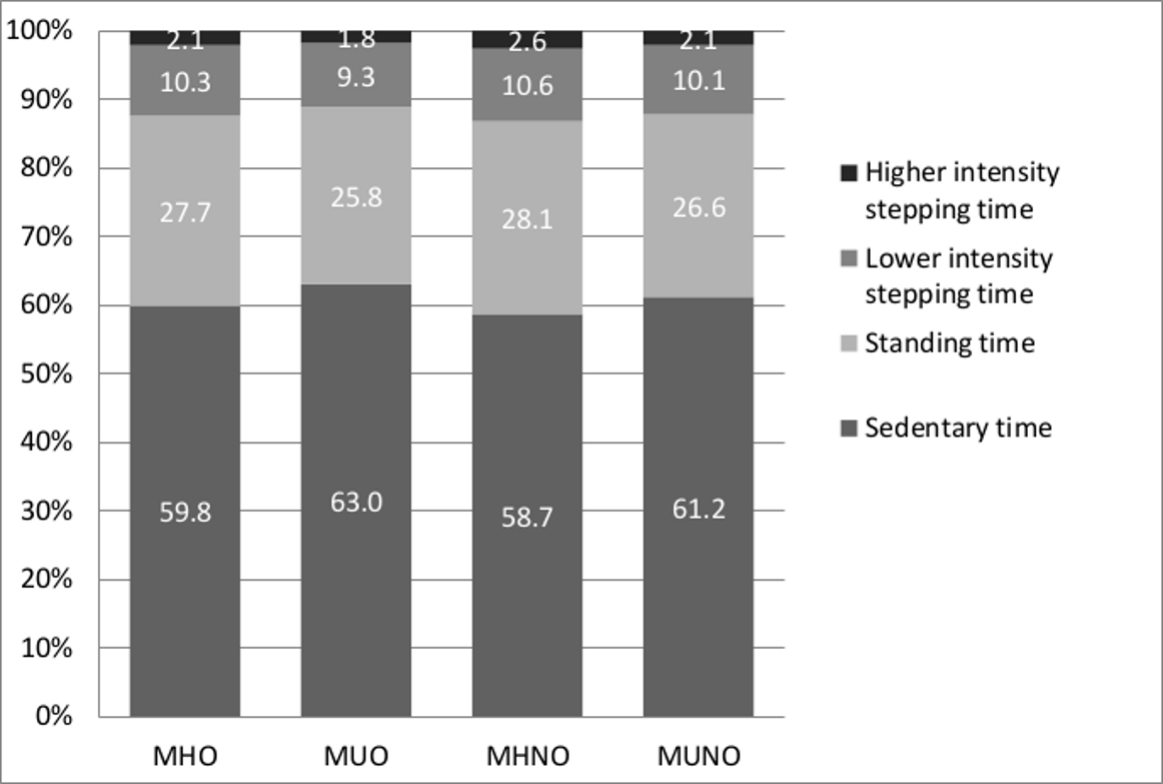
proportional sedentary, standing and stepping time (high and low intensity) per waking day, adjusted for age, sex, educational level, smoking, alcohol use, type 2 diabetes status, history of cardiovascular diseases and mobility limitation.

On average, individuals spent 60.1% of total daily waking time sedentary, 27.3% standing and 12.6% stepping. Fig 1 shows the mean percentage sedentary, standing and stepping time (high intensity and low intensity) of daily waking time for each group, adjusted for age, sex, educational level, smoking status, alcohol use, waking time, T2DM, history of CVD and mobility limitation. Overall, the highest levels of sedentary time per day and the lowest levels of stepping time per day were found in the MUO while the MHNO were least sedentary and most active. MUO and MUNO spent more daily waking time sedentary (63.0% and 61.2%) compared to MHO and MHNO (59.8% and 58.7%), and less time standing (25.8% and 26.6% versus 27.7% and 28.0%) (Fig 1). The MUO spent per day less time stepping in both low and high intensity compared to the other groups.

Table 2 shows that, after adjustment for age, sex, educational level, smoking status, alcohol use, waking time, T2DM, history of CVD and mobility limitation, statistically significant differences in sedentary time per day were found between MHO and MUO (563.5 versus 593.0 min. per day, p = 0.02) and between MHNO and MUNO (553.3 versus 576.6 min. per day, p<0.01). These differences remained significant after adjustment for high intensity stepping time (step frequency ≥ 110 steps/min) (Model 3). Additionally, the MUNO were less sedentary than the MUO (576.6 versus 593.0 min. per day, p = 0.03) (Model 2), but this difference was no longer statistically significant after adjustment for high intensity stepping time (573.3 versus 586.4 min. per day, p = 0.12) (Model 3).

**Table 2:** Adjusted means (95%CI) of sedentary behavior and physical activity variables of the four groups with and without obesity and the metabolic syndrome.

	Obese		Non-obese		Pairwise differences, p-values					
	MHO	MUO	MHNO	MUNO	MHO-MUO	MHO-MHNO	MHO-MUNO	MUO-MHNO	MUO-MUNO	MHNO-MUNO
**Model 1**										
Sedentary time, minutes/day	562.0 (545.1–579.0)	604.7 (596.2–613.2)	547.8 (543.1–552.6)	581.6 (573.8–589.3)	**<0.01**	0.68	0.25	**<0.01**	**<0.01**	**<0.01**
Standing time, minutes/day	262.0 (248.7–275.3)	239.1 (232.5–245.8)	267.2 (263.5–270.9)	248.3 (242.2–254.4)	**0.02**	1.00	0.41	**<0.01**	0.26	**<0.01**
Total stepping time, minutes/day	118.8 (111.6–126.0)	99.0 (95.4–102.6)	127.8 (125.8–129.8)	113.0 (109.7–116.3)	**<0.01**	0.11	0.90	**<0.01**	**<0.01**	**<0.01**
Low intensity stepping time, minutes/day	98.9 (92.9–104.9)	83.6 (80.6–86.6)	101.8 (100.1–103.5)	93.8 (91.1–96.5)	**<0.01**	1.00	0.80	**<0.01**	**<0.01**	**<0.01**
High intensity stepping time, minutes/day	14.1 (12.0–16.5)	9.8 (9.1–10.6)	19.1 (18.3–20.0)	13.7 (12.8–14.8)	**<0.01**	**<0.01**	1.00	**<0.01**	**<0.01**	**<0.01**
Sedentary breaks count/day*	50.9 (48.3–53.4)	51.1 (49.8–52.3)	56.1 (55.4–56.8)	55.0(53.9–56.2)	1.00	**<0.01**	**0.02**	**<0.01**	**<0.01**	0.79
**Model 2**										
Sedentary time, minutes/day	563.5 (546.8–580.3)	593.0 (584.1–602.0)	553.3 (548.4–558.2)	576.6 (568.8–584.4)	**0.02**	1.00	1.00	**<0.01**	**0.03**	**<0.01**
Standing time, minutes/day	261.1 (247.9–274.4)	244.6 (237.5–251.7)	264.6 (260.7–268.5)	250.8 (244.6–257.0)	0.19	1.00	1.00	**<0.01**	1.00	**<0.01**
Total stepping time, minutes/day	118.2 (111.1–125.3)	105.2 (101.4–109.0)	125.0 (122.9–127.0)	115.4 (112.2–118.7)	**0.01**	0.42	1.00	**<0.01**	**<0.01**	**<0.01**
Low intensity stepping time, minutes/day	98.4 (92.5–104.3)	87.7 (84.5–90.9)	99.9 (98.2–101.7)	95.5 (92.7–98.3)	**0.01**	1.00	1.00	**<0.01**	**<0.01**	0.06
High intensity stepping time, minutes/day	13.9 (12.0–16.2)	11.5 (10.6–12.5)	17.9 (17.1–18.7)	14.6 (13.5–15.6)	0.18	**0.02**	1.00	**<0.01**	**<0.01**	**<0.01**
Sedentary breaks count/day *	50.9 (48.4–53.5)	51.3 (50.0–52.7)	56.0 (55.3–56.7)	55.0 (53.8–56.1)	1.00	**<0.01**	**0.03**	**<0.01**	**<0.01**	1.00
**Model 3**										
Sedentary time, minutes/day	560.0 (543.8–576.2)	586.4 (552.1–580.8)	556.9 (552.1–580.8)	573.3(565.7–580.8)	**0.03**	1.00	0.90	**<0.01**	0.12	**<0.01**

Model 1: adjusted for age, sex, educational level, smoking, alcohol use, waking time. Model 2: model 1 + adjustment for T2DM status, history of CVD and mobility limitation. Model 3: model 2 + adjustment for high intensity stepping time.

*additionally adjusted for sedentary time

P-values in bold were statistically significant (α<0.05).

Total stepping time per day was statistically significantly higher in MHO compared to MUO (118.2 min. versus 105.2 min. per day; p<0.01), which was caused by a significant difference in low intensity stepping time (98.4 versus 87.7 min. per day, p = 0.01) (Model 2). MHNO spent significantly more total stepping time per day compared to MUNO (125.0 versus 115.4 min. per day; p<0.01), which was due to a significant difference in high intensity stepping time (17.9 versus 14.6 min. per day, p<0.01) (Model 2). Additionally, both low and high intensity stepping time per day was lower in MUO compared to MUNO (87.7 versus 95.5 min. per day, p<0.01; 11.5 versus 14.6 min. per day, p<0.01) (Model 2). Daily standing time statistically significantly differed between MHNO and MUNO (264.6 versus 250.8 min. per day, p<0.01). The mean number of sedentary breaks per day was statistically significantly higher in MHNO compared to MHO (56.0 versus 50.9 per day; p<0.01), higher in MUNO compared to MUO (55.0 versus 51.3 per day; p<0.01), and higher in MUNO compared to MHO (55.0 versus 50.9; p = 0.03) (Model 2).

In additional analyses, similar results in physical activity (high and low intensity stepping time and standing time) and sedentary behavior (sedentary time and sedentary breaks) were found across the four groups when excluding all individuals with T2DM from our analysis. To compare differences in physical activity and sedentary behavior between the metabolically healthy and unhealthy participants within the obese and non-obese groups, we additionally adjusted for BMI; no alterations in the results were found (not tabulated). Further, using mean 24-h ambulatory blood pressure to define MetS instead of office blood pressure did not alter our findings (not tabulated). No significant interaction effects were found between sex and sedentary behavior and physical activity. Also, no significant interaction was found between obesity and MetS for any of the physical activity and sedentary behavior variables.

## Discussion

In the present study, we examined cross-sectional differences in objectively measured physical activity and sedentary behavior across groups of metabolically healthy and metabolically unhealthy obese and non-obese individuals. We found that, overall, the metabolically unhealthy obese (MUO) spent less time stepping and more time sedentary per day, while the metabolically healthy non-obese (MHNO) were the most physically active and the least sedentary. The physical activity and sedentary behavior patterns of the metabolically healthy obese (MHO) and the metabolically unhealthy non-obese (MUNO) were very comparable. In general, the metabolically healthy groups spent daily less time sedentary and more time stepping compared to the metabolically unhealthy groups. The differences in sedentary time per day across the groups were independent of high intensity stepping time. Further, the obese spent the lowest amount of time in high intensity physical activity per day and had the least number of sedentary breaks per day compared to non-obese, independent of the presence of metabolic syndrome (MetS). Our results suggest therefore that obesity as well as MetS were independently of each other associated with both physical activity (low and high intensity stepping) and sedentary time.

Our results are partly supported by a few studies comparing objectively measured physical activity in MHO and MUO. In line with our findings, Bell and colleagues [26] found that total physical activity was significantly higher in MHO compared to MUO. Similarly, Poelkens and colleagues [4] found a higher total step count in MHO compared to the MUO. Other researchers did also find a higher step count and total energy expenditure in MHO compared to MUO, but this was not statistically significant [24,25]. In line with the study of Bell and colleagues [26], using objectively measured physical activity data, and some studies using self-reported physical activity data [5,29], our study did not show any differences in high intensity stepping time as a measure for MVPA between MHO and MUO. In contrast, other researchers found self-reported MVPA to be higher in MHO compared to MUO [3,13,18–21]. Similar conflicting results were found in studies comparing self-reported MVPA in MHNO and MUNO [3,5,19,21,30]. We have previously shown that an extra hour of sedentary time was associated with and 39% increased odds for the metabolic syndrome, independent of high intensity stepping time [12]. In the current study we found that the MUO spent significantly more time sedentary compared to the MHO and MUNO were more sedentary than the MHNO. In line with our findings, higher levels of self-reported sedentary time have been found in MUNO compared to MHNO [23]. Conversely, other studies did not find any differences in self-reported sedentary behavior between MHO and MUO [18,23].

Previous literature has drawn particular attention to the study of metabolically healthy obesity and implies that not all obese individuals are metabolically similar. However, it is currently not fully understood how these differences can be explained. The development of obesity is due to a combination of physical inactivity, increased food intake, genetic factors and perhaps behavioral and environmental alterations [31]. Evidence suggests that fat distribution and inflammation play a role in the presence of MetS in obese individuals [4,24,32,33]. Importantly, visceral adipose and liver tissue mass are associated with the presence of MetS, insulin resistance and cardiovascular diseases, whereas subcutaneous adipose tissue may not [34–38]. Further, a growing body of literature indicates that obese individuals who are physically fit (i.e. fat and fit), have a similar cardiovascular risk as healthy non-obese individuals, because exercise can result in decreased cardio metabolic risk and positive changes in body composition [20,39]. Our study showed that MHO were more physically active, as shown by on average 13 minutes more stepping time per day, and were on average 26 minutes less sedentary compared to MUO. Low levels of physical activity and high levels of sedentary time may cause metabolic disturbances and may therefore explain why some individuals are metabolically healthy and others are not. Further, a more active lifestyle could be protective against future cardiovascular complications in those who are metabolically healthy. Future studies are warranted to examine the effect of physical activity throughout the life course on MetS and associated health outcomes.

The main strengths of the current study include its unique design. To our knowledge, this is one of the first studies investigating objectively measured physical activity across these four groups, and the first study investigating objectively measured sedentary behavior across these groups. Earlier studies mostly compared physical activity sedentary behavior patterns within obese (MHO versus MUO) or non-obese (MHNO versus MUNO) individuals. This study additionally allowed for cross-comparison between obese and non-obese groups, which revealed similarities between MHO and MUNO and additional differences between MUO and MUNO. Moreover, the majority of existing evidence in this field is based on self-reported physical activity and sedentary behavior, leading to less precise estimations of physical activity and sedentary behavior [40]. In the current study, physical activity and sedentary behavior were objectively measured continuously for seven days resulting in a comprehensive reflection of daily activity in free living conditions. Another strength of the current study is the large sample of both obese and non-obese individuals.

This study also has some limitations. Causal evidence of the current study is limited because of its cross-sectional design. It is therefore possible that the metabolically unhealthy individuals were less physically active as a result of their poorer health. This issue was partly addressed by adjusting for T2DM status, CVD and mobility limitation. However, prospective studies are needed to confirm our findings. In addition, mediation analysis could potentially elucidate to what extent physical activity and sedentary behavior contribute to the presence of MetS in obese and non-obese individuals. Further, The Maastricht Study population contains an oversampling of individuals with T2DM. This leads to an overrepresentation of T2DM in the groups with metabolically unhealthy individuals, which could have led to an overestimation of the differences between healthy and unhealthy groups. However, we adjusted for T2DM in all analysis, and in additional analyses excluding individuals with T2DM our results were similar. Further, the BMI range in the non-obese was broader; additional adjustment for BMI within the obese and non-obese revealed similar differences in sedentary behavior and physical activity between the healthy and unhealthy groups. Finally, we did not address dietary intake as a potential confounder in our analyses. Further research into diet across these groups may further explain the presence of MetS in obese as well as in non-obese individuals.

## Conclusion

In conclusion, levels of physical activity and sedentary behavior may contribute to the explanation why some individuals are metabolically healthy and others are not. Hence, longitudinal investigations are needed to confirm our findings. While in general the obese are less physically active and more sedentary than the obese, particular attention needs to be paid to the MUO as they have shown to be the most sedentary and least physically active. In addition, MUNO could be a novel target group for lifestyle interventions in the non-obese population. Physical activity intervention programs with a particular focus on MUO as well as MUNO may be important and could potentially reduce the associated health consequences in these groups.
